# Retrospective longitudinal study of ALS in Cyprus: Clinical characteristics, management and survival

**DOI:** 10.1371/journal.pone.0220246

**Published:** 2019-09-06

**Authors:** Christiana A. Demetriou, Petros M. Hadjivasiliou, Kleopas A. Kleopa, Yiolanda P. Christou, Eleni Leonidou, Theodoros Kyriakides, Eleni Zamba-Papanicolaou

**Affiliations:** 1 Department of Primary Care and Population Health, University of Nicosia Medical School, Nicosia, Cyprus; 2 Neurology Clinic D, The Cyprus Institute of Neurology and Genetics, Nicosia, Cyprus; 3 The Cyprus School of Molecular Medicine, The Cyprus Institute of Neurology and Genetics, Nicosia, Cyprus; 4 Neurology Clinic E, The Cyprus Institute of Neurology and Genetics, Nicosia, Cyprus; 5 Neurology Clinic C, The Cyprus Institute of Neurology and Genetics, Nicosia, Cyprus; 6 Neurology Clinic A, The Cyprus Institute of Neurology and Genetics, Nicosia, Cyprus; Clinical Research on Neurological Diseases, CHINA

## Abstract

**Introduction:**

Amyotrophic lateral sclerosis (ALS) is a rare, progressive neurodegenerative disease. There is heterogeneity of clinical phenotypes while a clinical characterization of ALS in Cyprus is still lacking. The aim of this 30-year retrospective study of ALS in Cyprus is to determine the demographic characteristics of patients, the clinical features of the disease, the uptake of supportive therapies and factors influencing survival.

**Methods:**

All ALS patients seen at the Cyprus Institute of Neurology and Genetics from January 1985 until July 2015 were included. Medical records of eligible patients were used for data extraction and compilation of an ALS database. Clinical features were compared between gender categories using univariate tests, while survival was assessed using Kaplan-Meier curves. Cox proportional hazards models were used to identify prognostic factors for survival.

**Results:**

One hundred and seventy-nine ALS patients were included in the study, of whom 7 had a positive family history. Most clinical characteristics of ALS did not differ from what is observed in other European countries. However, some clinical characteristics were unique to our population, such as an increased acceptability and utilisation of supportive treatments such as gastrostomy.

**Conclusions:**

Overall, clinical characteristics of patients with ALS in the Republic of Cyprus do not differ from other European counties. Our study demonstrates a high acceptance and utilisation of supportive interventions enhancing survival, in the context of a multidisciplinary approach offered in the single tertiary centre that services the whole Cypriot ALS population. The findings of this paper are of value to the health professionals treating ALS in Cyprus.

## Introduction

Amyotrophic lateral sclerosis (ALS) is the most common form of adult-onset motor neuron disease and the third most common neurodegenerative disease[1].

Heterogeneity of clinical phenotypes is well recognized, but clinically classical ALS is characterized by progressive weakness over time and space and the coexistence of upper (UMN) and lower (LMN) motor neuron signs encompassing multiple body regions[2].

The majority of ALS patients present with limb-onset disease (65–75%), with preferential wasting and weakness of the thenar muscles[3]. Limb-onset ALS is typically very slowly progressive[4]. ‘Bulbar-onset’ ALS first affects the muscles of speech, swallowing and mastication. It describes 20% of ALS cases[2] and is commonly associated with disordered affect, cognition and emotional lability[5]. Compared with limb-onset disease where there is male predominance, there is female predominance in bulbar-onset disease[4].

Despite its phenotypical heterogeneity, ALS is universally fatal since paralysis that progresses rapidly leads to respiratory failure and death within two to three years of symptom onset. ALS patients display the shortest median survival among neurodegenerative disorders[6,7]. The backbone of ALS management is symptomatic treatment and palliative care.

Ten percent of ALS are classified as familial in nature (FALS), where the disease is inherited in Mendelian mostly dominant manner, while the remaining 90% are considered sporadic (SALS) with no family history of the disease[1]. Phenotypically, FALS cases and SALS cases are essentially indistinguishable.

Incidence and prevalence of ALS display a high variability. The variability can be somewhat attributed to differences in study design, or may reflect true demographic and/or geographic differences [8,9]. In Cyprus, average annual crude incidence over the past 25 years was 1.26 cases per100,000 person-years. Prevalence of ALS was 7.9 cases per 100,000 population at the beginning of 2015[10]. Incidence and prevalence both increased over the past 25 years, and the increase in incidence rates remained even after age-standardization[10].

Despite the previous detailed exploration of ALS epidemiology in Cyprus by our group[10], a clinical investigation of ALS in Cyprus is still lacking. The aim of this project is to determine the clinical presentation of ALS patients, the uptake of supportive therapies, and factors associated with survival, over the past 30 years in Cyprus.

## Material and methods

### Patient cohort

This study is a retrospective clinical investigation of previously identified[10] ALS patients seen at the Cyprus Institute of Neurology and Genetics (CING) between 1 January 1985 and 31 July 2015. Recruitment details of patients are outlined elsewhere[10]. Briefly, cases were eligible for inclusion in the study if they met the El Escorial criteria (EEC) of definite or probable ALS[11–13], were of Cypriot nationality and resided in the Republic of Cyprus. A total of 179 met these inclusion criteria and were thus clinically investigated. In Cyprus, CING is the only tertiary referral centre for neurological conditions and the vast majority of ALS patients are referred there for clinical care. For this and additional reasons outlined in the discussion, a near complete capture is expected. This clinical investigation was approved by the Cyprus National Bioethics Committee, which waived the requirement for informed consent since accessed data were anonymized.

### Collection and organization of data

Medical records of eligible patients, from the clinical data bank of the CING, were used for detailed clinical data extraction to update CING’s ALS database. Specific clinical variables extracted from patients’ files are listed in S1 Table. The ALS database was updated regularly to reflect the patients’ status at their most recent clinical appointment. Date of death or end of data collection/end of study marked the end of follow-up.

### Statistical analysis

Univariate tests were used to assess clinical features between gender categories. The same tests were also used to compare the uptake of advanced directives between cases diagnosed in two different time-periods. The year of the publication of EFNS’s guidelines for optimal clinical approach to ALS[14], namely 2005, was used as the cut-off for the two time-periods.

Kaplan-Meier (KM) life table analysis was used to assess survival. Survival Kaplan Meier curves were constructed to visualize survival by gender, age at diagnosis (under 65 vs. 65+), age at onset (below or above median 59 years), time period of diagnosis (before *vs*. after 2005), diagnostic delay (below or above median 11 months), tracheostomy (yes vs. no), PEG feeding (yes vs. no), and first symptom at onset (for symptoms with n>5). Cox regression was used to assess the effect of each of these variables on survival, independently. Time was measured in months from diagnosis to death, loss to follow-up or end of study as suggested by Rooney et al (2013)[15].

Cox proportional hazards analyses were also used to simultaneously model multiple factors associated with prognosis, since some clinical characteristics are inter-related. Entries with missing values were dropped prior to modelling. All variables were initially included in the model, and non-significant variables were then sequentially removed from the model via backwards elimination. Successive models were compared using likelihood ratio testing with P<0.05 as the significance threshold. Further to the selection of the best fit model, routine diagnostics were carried out to ensure model validity.

All analyses were carried out using STATA version SE12.

## Results

### Clinical features

ALS patients’ clinical characteristics, as a total and stratified by gender, are presented in Table 1. As previously reported[10], there was a male predominance with a gender ratio of 1.45 M:F (106/73), disease onset was at a mean age of 58.6±10.9 years, diagnosis was at a mean age of 59.8±10.6, and only 4% (n = 7) of our sample had a family history of ALS. Clinical characteristics did not differ between gender categories (Table 1).

**Table 1:** Clinical characteristics of ALS patients, by gender category.

			Gender		
Characteristic	Statistic	Total	Males	Females	P-value (test)*
	Count	179	106	73	
**Age at Diagnosis**^**¥**^	Mean (SD)	59.8 (10.6)	59.8 (11.0)	59.9(10.1)	0.983 (ANOVA)
**Age at Symptom Onset**^**¥**^	Mean (SD)	58.6 (10.9)	58.7(11.1)	58.5(10.6)	0.875 (ANOVA)
**Diagnostic delay from Symptom onset to Diagnosis (months)**^**¥**^	Mean (SD)	13.6 (12.8)	13.7(13.6)	13.6 (11.7)	0.547 (Wilcoxon rank-sum)
**Family History of ALS**^**¥**^					
- **Yes**	Count(%)	7 (3.9)	5 (4.7)	2 (2.8)	0.704 (Fisher’s exact)
- **No**		170 (95.0)	101 (95.3)	69 (94.5)	
- **Information Missing**		2 (1.1)	0 (0.0)	2 (2.7)	
**Body region first affected**					
- **Spinal**	Count(%)	146 (81.6)	90 (84.9)	56 (76.7)	0.179 (Chi-squared)
- **Bulbar**		33 (18.4)	16 (15.1)	17 (23.3)	
**First Symptom at Onset**					
- **Dysarthria**	Count(%)	30 (16.8)	15 (14.2)	15 (20.5)	0.295 (Fisher’s exact)
- **Impaired Balance**		1 (0.6)	0 (0.0)	1 (1.4)	
- **Limb Weakness**		117 (65.4)	72 (68.0)	45 (61.6)	
- **Muscle Cramps**		14 (7.8)	10 (9.4)	4 (5.5)	
- **Muscle Stiffness**		11 (6.1)	5 (4.7)	6 (8.2)	
- **Muscle Twitching**		3 (1.7)	3 (2.8)	0 (0.0)	
- **Truncal Weakness**		1 (0.5)	0 (0.0)	1 (1.4)	
- **Dysphagia**		2 (1.1)	1 (0.9)	1 (1.4)	
**Respiratory Problems**					
- **Yes**	Count(%)	162 (90.5)	97 (91.5)	65 (89.1)	0.742 (Fisher’s exact)
- **No**		10 (5.6)	7 (6.6)	3 (4.1)	
- **Information Missing**		7 (3.9)	2 (1.9)	5 (6.8)	
**Time from Diagnosis to respiratory symptoms if they did develop (months)**	Median (IQR)	12.0 (21.0)	11.0 (19.0)	13.0 (24.0)	0.276 (Wilcoxon rank-sum)
**Tracheostomy**					
- **Yes**	Count(%)	32 (17.9)	22 (208)	10 (13.7)	0.269 (Chi-square test)
- **No**		147 (82.1)	84 (79.2)	63 (86.3)	
**Time from Diagnosis to tracheostomy (months)**	Median (IQR)	21.0 (26.0)	24.0 (21.0)	19.5 (38.0)	0.642 (Wilcoxon rank-sum)
**Dysphagia**					
- **Yes**	Count(%)	156 (87.2)	92 (86.8)	64 (87.7)	0.287 (Fisher’s exact)
- **No**		16 (8.9)	12 (11.3)	4 (5.5)	
- **Information Missing**		7 (3.9)	2 (1.9)	5 (6.8)	
**Time from Diagnosis to dysphagia if it did develop (months)**	Median (IQR)	7.0 (17.0)	7.0 (16.5)	6.5 (17.0)	0.605 (Wilcoxon rank-sum)
**PEG feeding**					
- **Yes**	Count(%)	61 (34.1)	37 (34.9)	24 (32.9)	0.930 (Chi-squared)
- **No**		119 (65.9)	69 (65.1)	49 (67.1)	
**Time from Diagnosis to PEG (months)**	Median (IQR)	17.0 (18.0)	19.0 (21.0)	16.0 (12.0)	0.745 (Wilcoxon rank-sum)
**Cognitive Impairment**					
- **Yes**	Count(%)	18 (10.0)	12 (11.4)	6 (8.2)	0.577 (Chi-squared)
- **No**		152 (84.9)	91 (85.8)	61 (83.6)	
- **Information Missing**		9 (5.1)	3 (2.8)	6 (8.2)	
**Riluzole uptake**					
- **Yes**	Count(%)	151 (84.3)	88 (83.0)	63 (86.3)	0.577 (Chi-squared)
- **No**		28 (15.7)	18 (17.0)	10 (13.7)	
**Deceased**					
- **Yes**	Count(%)	127 (70.9)	80 (75.5)	47 (64.4)	0.255 (Chi-squared)
- **No**		52 (29.1)	26 (24.5)	26 (35.6)	
**Survival from Symptom onset to Death if deceased (months)**	Median (IQR)	39.0 (35.0)	36.5 (39.0)	40.0 (31.0)	0.525 (Wilcoxon rank-sum)
**Survival from Diagnosis to Death if deceased (months)**	Median (IQR)	27.0 (28.0)	25.5 (31.5)	27.5 (25.0)	0.761 (Wilcoxon rank-sum)
**2year survival from symptom onset**					
- **Yes**	Count(%)	141 (78.8)	82 (77.4)	59 (80.8)	0.187 (Chi-squared)
- **No**		31 (17.3)	22 (20.8)	9 (12.1)	
- **Information Missing**		7 (3.9)	2 (1.8)	5 (6.8)	
**5year survival from symptom onset**					
- **Yes**	Count(%)	79 (44.1)	46 (43.4)	33 (45.2)	0.580 (Chi-squared)
- **No**		93 (52.0)	58 (54.7)	35 (47.9)	
- **Information Missing**		7 (3.9)	2 (1.9)	5 (6.9)	

* p<0.05 taken as evidence for a statistical significant association

^¥^ Results also reported elsewhere **[10]**

One hundred and twenty-eight patients had a recorded date of death and 45 were still alive at the end of the study period.

**Fig 1:**
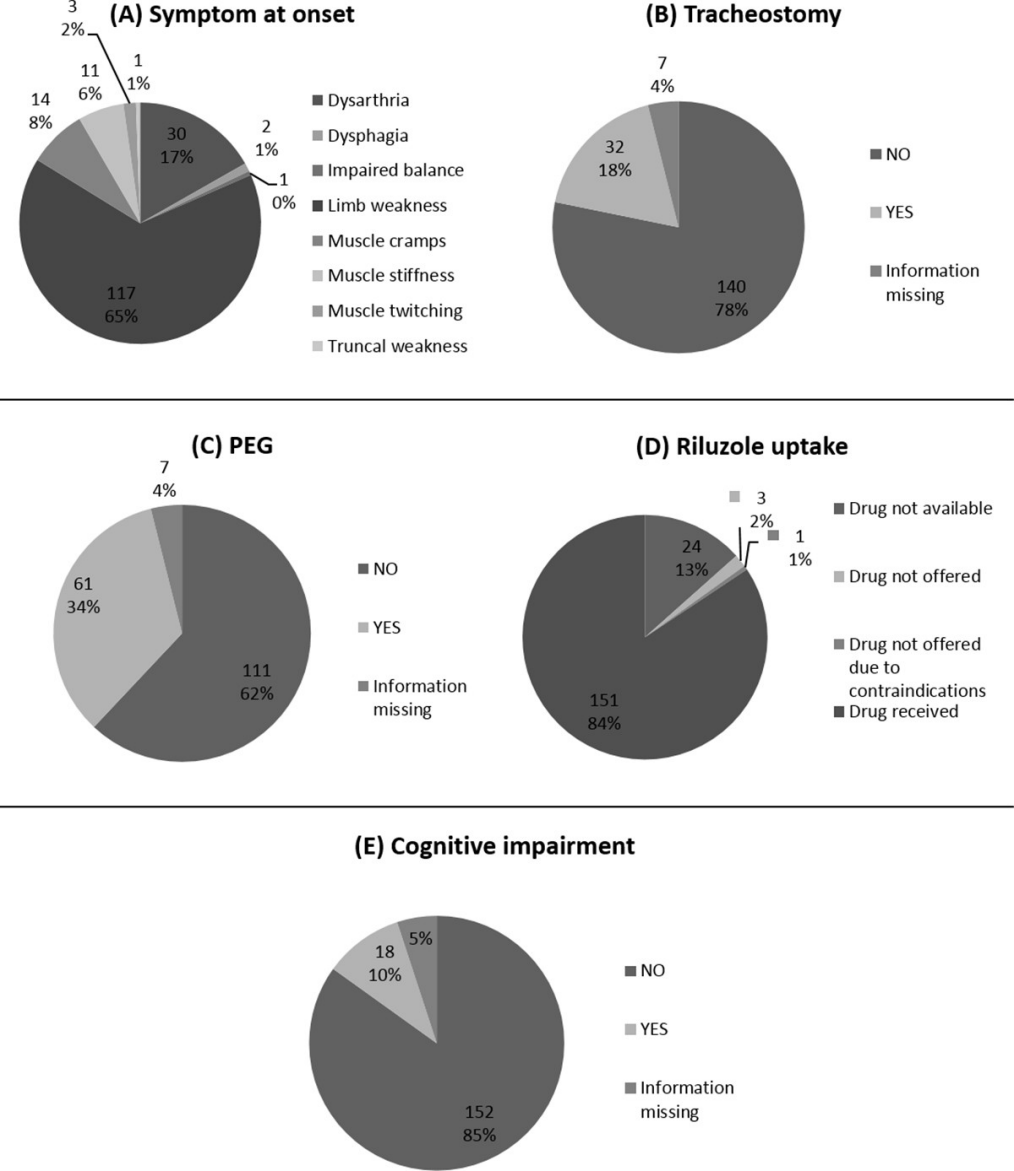
Clinical features of 179 patients with ALS. First symptom at onset (A), tracheostomy (B), PEG (C), riluzole uptake (D) and cognitive impairment (E).

Symptom and signs at diagnosis was available for all patients (Table 1). The majority of patients (81.6%) had a spinal onset with the remaining 18.4% having a bulbar onset. More specifically, 117 patients (65.4%) had limb weakness as their first symptom at onset, followed by 30 patients (16.8%) with dysarthria. Muscle cramps were the first sign in 14 patients (7.8%) and muscle stiffness in 11 (6.1%). Dysphagia, impaired balance, muscle twitching and truncal weakness were far less common as first symptom at onset (Fig 1A).

Respiratory symptoms developed in 90.5% of patients during the course of the disease, and of these, 32 patients (~18% of all patients) underwent tracheostomy (Fig 1B). Even though respiratory symptoms were equally common in both genders, a greater percentage of males underwent tracheostomy compared to females (20.8 vs. 13.7%). Almost equal percentages of males and females denied tracheostomy, despite recommendation (60.7% and 58.3, respectively) (Table 2). Comparison of the two time periods (1985–2004 *vs*. 2005–2014) demonstrated significant increases in the uptake of tracheostomy, significant decreases in the time period between diagnosis and onset of respiratory symptoms, and significant decreases in the median time from diagnosis to tracheostomy, despite similar prevalence of respiratory symptoms (S2 Table). Unfortunately, information on non-invasive mechanical ventilation (NIV) was not systematically reported in the patients’ medical files and thus no conclusions on or comparisons of its use could be made.

**Table 2:** Advanced directives and supportive management.

	All Patients		Males		Females	
	No. of patients	% of patients*	No. of patients	% of patients*	No. of patients	% of patients*
**PEG**						
** Insertion**	61	66.3	37	63.8	24	68.6
** Denial despite** **recommendation**	31	33.7	21	36.2	10	29.4
**Tracheostomy**						
** Insertion**	32	40.0	22	39.3	10	41.7
** Denial despite** **recommendation**	48	60.0	34	60.7	14	58.3
**PEG and Tracheostomy**						
** Insertion**	26	49.1	18	48.6	8	50.0
** Denial despite** **recommendation**	27	50.9	19	51.4	8	50.0

* % of patients to whom the respective directive was recommended.

Just as common was dysphagia, which developed in 87.2% of patients, with 34% (n = 61) of all patients undergoing elective gastrostomy placement (PEG feeding) (Fig 1C). Dysphagia was again equally common in males and females, and the percentage of male and female patients undergoing PEG insertion was similar (34.9 vs. 32.9, respectively) (Table 2). Dysphagia was also equally common in patients diagnosed before and after 2005. However, there was a significant decrease in the duration of time between diagnosis and the development of dysphagia, a non-significant decrease in the time from diagnosis to PEG insertion, and a significant increase in the uptake of PEG insertion, when comparing the two time-periods (S2 Table). Overall, the majority of patients with whom tracheostomy was discussed did not choose to undergo the procedure (60.0%), but the majority of patients to whom PEG was recommended, did proceed with the insertion (66.3%) (Table 2).

The median time and interquartile range (IQR) from diagnosis to development of respiratory symptoms was 12.0 (21.0) months and from diagnosis to tracheostomy 21.0 (26.0) months (Table 1). Dysphagia was an earlier event since it developed at a median time of 7.0 (17.0) months from diagnosis, and patients underwent PEG surgery at a median time of 17.0 (18.0) months after diagnosis. However, the large IQRs of these features indicate the variable nature of the disease and how symptom onset and medical interventions can differ substantially between patients.

Riluzole uptake was high among Cypriot patients since overall 84% of all patients received the drug (Fig 1D). Riluzole was introduced in Cyprus during 1995, therefore for patients who died up to 1995 (n = 25), the drug was not available. Of all patients surviving past 1995 or diagnosed after 1995 (n = 156), 96.8% took Riluzone. For the remaining patients, the drug was not prescribed either because their condition was already severely deteriorated in 1995 (1.3%) or due to contraindications, namely chronic renal failure (1.9%).

Lastly, only 10% of Cypriot patients with ALS exhibited cognitive impairment, mostly at advanced disease stages (Fig 1E). The criterion used for the diagnosis of cognitive impairment, in this cohort, was a score of ≤23 on the Mini-Mental State Examination. Only for one patient was there also imaging evidence of fronto-temporal degeneration.

### Survival

**Fig 2:**
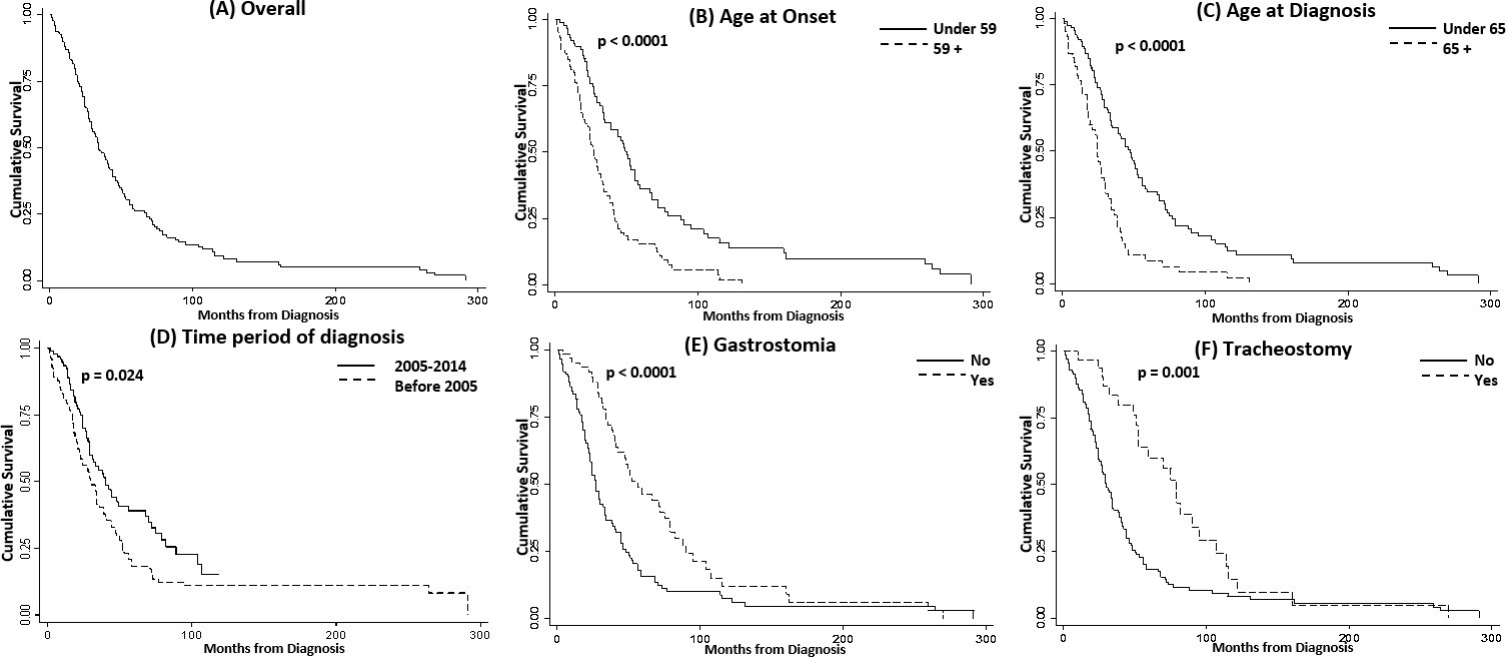
Kaplan-Meier survival estimates; overall and by specific demographic and clinical characteristics. Survival is measured in months from diagnosis. A) Overall survival for the whole cohort; B) by age at onset (under 59 vs. 59+ years) (p<0.0001); C) by age at diagnosis (under 65 vs. 65+) (p<0.0001); D) by time period at diagnosis (before 2005 vs. after)E) by PEG feeding (p<0.0001); F) by tracheostomy (p = 0.001).

Overall survival of the whole cohort from diagnosis is shown in Fig 2A (including 25% still alive at the end of data collection). Median survival from diagnosis was 27.0 (Range: 1.0–291.0) months and from symptom onset 39.0 (Range: 7.0–313.0). It is surprising that ~20% (37 patients) of the cohort survived longer than 60 months after diagnosis.

Survival by age at diagnosis (under 65 vs. 65+), by age at onset (under 59 vs. 59+ years), by time period at diagnosis (before 2005 *vs*. 2005–2014), by tracheostomy, and by PEG feedingis shown in Fig 2B–2F. In univariate analyses age at diagnosis (p<0.0001), age at onset (p<0.0001), time period of diagnosis (p = 0.024), tracheostomy (p = 0.001) and PEG (p<0.0001) were all significantly associated with survival. Gender, diagnostic delay, and first symptom at onset were not significantly associated with survival in univariate analyses (S1 Fig).

A multivariate Cox proportional hazards model was used to assess whether demographic or clinical variables together influenced survival (Table 3). In this model increasing age at onset was shown to be associated with decreased survival (_HRper year_:1.04, 95% CI: 1.02–1.06). On the contrary, diagnostic delay (HR_per month_: 0.97, 95% CI: 0.96–0.99), tracheostomy (HR_yes vs. no_: 0.37, 95% CI: 0.20–0.68) and PEG (HR_yes vs. no_: 0.52, 95% CI: 0.33–0.80) were associated with increased survival.

**Table 3:** Multivariate cox regression model of survival in amyotrophic lateral sclerosis, n = 172.

Parameter	HR	95% CI	p-value*
Tracheostomy			
- **No**	1.00		
- **Yes**	0.37	0.20–0.68	0.001
**PEG**			
- **No**	1.00		
- **Yes**	0.52	0.33–0.80	0.004
**Age at onset** **(per unit increase)**	1.04	1.02–1.05	<0.0001
**Diagnostic delay from symptom onset to diagnosis in months (per unit increase)**	0.97	0.96–0.99	0.002

* p<0.05 taken as evidence for a statistical significant association

## Discussion

A total of 179 cases of ALS were identified previously as part of an epidemiological investigation in Cyprus, which focused on incidence, prevalence and patient main demographic features[10].

This study of a reference centre population, is the first to describe the clinical phenotype of ALS in the Republic of Cyprus by investigating the clinical presentation, management and survival details of the previously identified ALS patients. For this investigation, the 30 year old clinical data base at the CING, a tertiary neurological referral centre, was interrogated, and ALS patients were retrospectively followed up from first symptom presentation until death or end of study.

Demographic characteristics of this patient cohort, such as a slight male predominance, a younger mean age at onset, and a 4% prevalence of familial cases were reported and discussed elsewhere[10].

In terms of clinical characteristics, Cypriot ALS patients were similar to patients of other nationalities. ALS is heterogeneous in site of disease onset, but the majority of ALS patients according to the literature present with spinal-onset disease (65–75%)[2,3,16]. Indeed, in this study, for both men and women spinal onset was more common (81.6%), and similar to the reported literature[4], bulbar onset was recorded more frequently in females than in males, 23.3% and 15.1% respectively.

Cognitive impairment during the course of the disease was rarely seen in these data compared with previous reports [17,18], but collection of data on this modality was not a focus of this study.

Aspects of nutritional and respiratory supportive care, like PEG and tracheostomy respectively, were examined as dysphagia and respiratory problems were found to present in almost 90% of all patients. Both of these supportive procedures, recommended following the EFNS guidelines[13,14], were found to be performed more often over the past 10 years, so that until the end of study period, 34% of patients have received PEG and 18% have undergone tracheostomy. Tracheostomy was more common in males, and a statistically significant male predominance in the use of mechanical ventilation was also reported elsewhere[19,20]. On the other hand, dysphagia and PEG insertion were similarly common in both genders. Unfortunately, no consistent information on the use of NIV is available for this cohort. However, as per the EFNS guidelines, NIV is recommended to all Cypriot patients following the onset of respiratory problems. Tracheostomy recommendation is reserved for when NIV is no longer able to maintain satisfactory oxygen saturation levels or for the rare cases when a patients cannot cooperate with NIV. Despite availability of these advanced directives for several years, their popularity has been increasing in Cyprus from 2000 and onwards.

Comparison of the cases diagnosed before and after 2005, demonstrated that the median time from diagnosis to respiratory symptoms, was significantly higher for the time-period prior to 2005, most likely due to less frequent patient follow-up by a multi-disciplinary team and thus perhaps a delayed ability to diagnose respiratory problems. In the past decade, however, time from diagnosis to respiratory symptoms seems to have levelled-off to around 11 months. In parallel, to the earlier onset of respiratory problems, the median time from diagnosis to tracheostomy has significantly decreased between the two time-periods.

With respect to dysphagia, the median time from diagnosis to dysphagia shows a significant decrease between before and after 2005. This might again reflect better and more frequent monitoring of this symptom in ALS patients by specialised health professionals. In parallel, the median time from diagnosis to PEG shows a considerable decrease through the time-periods, albeit non-significant, reflecting the EFNS guidelines for an earlier PEG insertion, even before the onset of respiratory problems[13,14].

According to the ALS CARE database, less than 5% of ALS patients in North America receive invasive mechanical ventilation. On the contrary, non-invasive mechanical ventilation is taken up by 21% of patients[21]. In Italy 10.6 to 31.3 percent of patients underwent tracheostomy[22–24], in France 2–5%[24,25], in the UK 6%[26]while, only 6.7% of men and 3.8% of women with ALS underwent tracheostomy in Sweden and Norway[19,27]. In contrast, 11% of Korean ALS patients, about 27–45% of Japanese and 21.0% of Taiwanese ALS patients underwent tracheostomy[28–31].

Our findings may indicate that cultural acceptability of Cypriots regarding tracheostomy is more similar to that of Asian, rather than Caucasian ethnicities, with the exclusion of Italians who are also more likely to undergo the procedure. Apart from the cultural differences, the fact that each country has a different health care system with differential practices and access to palliative care intervention, contributes to varying rates of adoption of these procedures across countries.

PEG is another underutilized recommendation in the US since 9% of patients in North America received PEG[21], much lower than the percentage (18.1%) reported about the Korean ALS patients[28]. Among Cypriot ALS patients, PEG insertion is even more highly accepted.

Consistent with the literature, most patients die on average 3 years after symptom onset, although a noteworthy proportion of patients survive beyond five years[32]. Some studies have delineated a difference in survival by gender, while others have not[33]. Here, the median survival time from symptom onset was shown to be higher for females than males (40 vs. 36.5 months respectively), albeit non-significant, and there was also a non-significant higher probability of females being alive both two and five years after symptom onset. This observation was surprising given the larger percentage of bulbar onset ALS among females (which is associated with worse prognosis[7]) and the smaller percentage of females choosing to undergo tracheostomy. However, investigation in larger numbers is necessary to delineate whether this finding is a chance finding or a true difference in the Cypriot ALS population.

Positive effects on survival were demonstrated for the use of PEG and/or tracheostomy, and a longer time from symptom onset to diagnosis. The increased utilisation of the supportive invasive interventions, as well as the prolonged survival have been associated to the provision of multidisciplinary care itself in previous studies[34–37]. The association of longer time from symptom onset to diagnosis with a better survival was again consistent with the results of previous studies [7,38]. One possible explanation for this association might be that longer time from symptom onset to diagnosis correlates to a milder phenotype with a not so rapidly progressive course. On the other hand, poor prognosis was associated with a later age at disease onset. Older age at diagnosis was repeatedly shown to be the strongest predictor of poor survival in other studies[7,39,40].

Invasive mechanical ventilation has been clearly found effective in relieving chronic hypoventilation and prolonging life in ALS patients in other studies as well[22,41]. However, only few recent studies have shown a survival benefit from PEG[23,42,43]. A reason for the conflicting results in the literature might be the timing of PEG insertion, since late PEG insertion may not show any survival benefit[32,42]. The fact that in our study, there is a survival benefit, may point to the timely recommendation and insertion of PEG in Cypriot ALS patients, supporting the positive role of palliative care within the coordinated inter-professional ALS care as shown in other studies[36,44].

As expected, there was a survival benefit in those diagnosed in the time period 2005–2014 compared to before 2005. This benefit is most likely explained by the increased uptake of advanced directives since adjusting for these factors eliminated significance. Riluzole was given to all patients who survived past 1995 or were diagnosed after 1995 and did not have any contraindications for use of this drug. Therefore a survival analysis of this clinical variable was of little interest, since any survival benefit seen for Riluzole would be a reflection of the advances in ALS care over the years or of the absence of contraindications such as chronic renal failure.

The main strength of this investigation is the almost complete ascertainment of ALS cases. Case ascertainment is expected to be very high because CING is the only tertiary neurological referral centre on the island, and, if not all, the vast majority of ALS cases are referred there for treatment. CING is accessible to all ALS patients since, in Cyprus, care costs are subsidized by the government for patients with chronic diseases including neurological disorders. Furthermore, the process of referral to CING is simple for ALS patients, requiring only a letter by their secondary physician. This referral system applies to all ages and has not changed over the years. Further, CING is located in the island’s capital which is easily accessible from all districts of Cyprus. Attending CING is not difficult, even for ALS patients, because families in Cyprus are very involved in the health of their members. This results in higher rates of attendance and more frequent evaluations compared to reported access to specialized ALS services offered in other countries[44,45]. Lastly, case ascertainment is expected to be near complete because government reimbursement for purchasing assistive technologies is available to patients only through CING processes.

A second strength of this study is the comprehensiveness, quality and recency of the information collected, since CING files are regularly updated from a multidisciplinary team of health specialists. Therefore, these results are based on complete or near-complete case ascertainment, rigorous follow-up and high-quality information allowing for comparisons with other similar studies.

However, even though the vast majority, if not all, Cypriot ALS patients are cared for at the CING, one cannot ignore the fact that this is a reference centre population and might, in some respects, differ from the general ALS population of the country. In addition, like other epidemiological studies of ALS, this study may suffer from poor phenotype definition, particularly prior to 1994 when the EEC criteria were introduced. Since this study included incident cases prior to 1990 until 2015, it spanned the publication dates of the 1994 'El Escorial' diagnostic criteria and the revised 2000 'Airlie House' criteria developed by the World Federation of Neurology. For as long as there is no definite test/marker to confirm ALS, the validity of the diagnosis is dependent on the clinical judgment and experience of the attending clinician[46]. However, following the establishment of CING, the diagnosis of patients by a few neurologists with a high level of expertise in ALS ameliorates this issue, surpassing poor phenotype definition[10].

This study provides the first clinical characterization of ALS in the Cypriot population, summarizing important clinical aspects of disease management for healthcare and other stakeholders in Cyprus. However, it also supports further investigation to unravel the genetic factors behind the FALS cases and the apparently sporadic ALS cases. The classification of ALS cases as familial or sporadic retains clinical utility, however it is questioned if sporadic ALS exists as a valid concept, so this classification should not be viewed in absolute terms[47]. This has obviously implications for the clinical care, genetic counselling and testing in ALS.

Based also to the growing notion that a genetic component underlies all ALS cases, genetic research is mandatory in an effort to unravel the genetic susceptibility underlying the identified Cypriot ALS patients. This genetic susceptibility could explain why the Cypriots are affected by the disease on average at a younger adult age compared to most of the other populations studied. It would be also interesting to find out the true burden of the most common genetic cause of ALS in Caucasians (the pathogenic C9ORF72 repeat expansion) on the Cypriot ALS population.

## Conclusion

In this first clinical study of ALS in Cyprus, phenotypic characteristics were found to be similar to those of other European countries. However, some observations such as a non-significantly increased survival in females compared to males as well as an increased acceptability of invasive procedures such as gastrostomy and tracheostomy differ somewhat from those described in the literature and warrant further investigation. Furthermore, our study supports previous evidence that specialized coordinated care in ALS delivered by a multidisciplinary centre can lead to high utilisation of supportive interventions and a subsequent survival benefit. Overall, these findings are of value to the health professionals providing evidence-based care to ALS patients in Cyprus and supports the important role of a multidisciplinary approach in improving the care that addresses the complex needs of the patients and their families.

## Supporting information

S1 Table Clinical variables extracted from patients’ files and investigated in this study. (DOCX)

S2 Table Clinical features and process of care of ALS patients in the Republic of Cyprus categorized by time-period of diagnosis. (DOCX)

S1 Fig Kaplan-Meier survival estimates of non-significant demographic and clinical characteristics. Survival is measured in months from diagnosis. A) by gender (male vs. female) (p = 0.654); B) by diagnostic delay (under 11 vs. 11+ months) (p = 0.066); C) by first symptom at onset (p = 0.677).
(DOCX)
