# Supplementary material for: Retrospective longitudinal study of ALS in Cyprus: Clinical characteristics, management and survival
Source: PLoS One. 2019 Sep 6;14(9):e0220246. doi: 10.1371/journal.pone.0220246 (PMC6730913; doi:10.1371/journal.pone.0220246)
Supplement: S1 Table — (DOCX) [file pone.0220246.s001.docx]

**S1 Table: Clinical variables extracted from patients’ files and investigated in this study**

| Clinical Presentation |
| --- |
| - Age at symptom onset - Site of onset - limb vs. bulbar - Diagnostic delay (time from onset to diagnosis) - Year of diagnosis |
| Clinical Course |
| - Respiratory symptoms - Dysphagia symptoms - Survival |
| Medical Comorbidities |
| - Cognitive impairment (assessed by the MMSE, scores≤23) |
| Family History |
| - Family history of ALS |
| Use of Therapies and Interventions |
| - Riluzole uptake - Tracheostomy and non-invasive mechanical ventilation - Gastrostomy |

Abbreviations: MMSE, mini-mental state examination
