# Supplementary material for: Retrospective longitudinal study of ALS in Cyprus: Clinical characteristics, management and survival
Source: PLoS One. 2019 Sep 6;14(9):e0220246. doi: 10.1371/journal.pone.0220246 (PMC6730913; doi:10.1371/journal.pone.0220246)
Supplement: S2 Table — (DOCX) [file pone.0220246.s002.docx]

S2 Table: Clinical features and process of care of ALS patients in the Republic of Cyprus categorized by time-period of diagnosis

|  |  | Time Period | | |  |
| --- | --- | --- | --- | --- | --- |
| Characteristic | **Statistic** | **1985-2004** | **2005-2014** | **P-value**  **(test)** | |
|  | **Count** | **84** | **95** |  | |
| Respiratory Problems   - Yes - No | Count  (%)* | 75 (87.2)  3 (3.5) | 88 (91.7)  7 (7.3) | 0.512  (Fisher’s Exact) | |
| Time from Diagnosis to respiratory symptoms (months) | Median (IQR) | 15.0  (24.0) | 10.5  (19.0) | 0.015  (Kruskal-Wallis) | |
| Tracheostomy   - Yes - No | Count  (%)* | 7 (8.1)  71 (82.6) | 25 (26.0)  70 (73.0) | 0.005  (Fisher’s Exact) | |
| Time from Diagnosis to tracheostomy (months) | Median (IQR) | 46.0  (86.0) | 19.5  (22.0) | 0.042  (Kruskal-Wallis) | |
| Dysphagia   - Yes - No | Count  (%)* | 71 (82.6)  7 (8.1) | 86 (89.6)  9 (9.4) | 1.000  (Fisher’s Exact) | |
| Time from Diagnosis to dysphagia (months) | Median (IQR) | 11.0  (21.0) | 4.0  (13.0) | 0.004  (Kruskal-Wallis) | |
| PEG   - Yes - No | Count  (%)* | 12 (14.0)  66 (76.7) | 50 (52.1)  45 (46.9) | <0.0001  (Fisher’s Exact) | |
| Time from Diagnosis to PEG (months) | Median (IQR) | 21.0  (39.0) | 16.0  (16.0) | 0.202  (Kruskal-Wallis) | |

*Failure of percentage values to add up to 100 denotes missing values
