# Supplementary figures and images for: Retrospective longitudinal study of ALS in Cyprus: Clinical characteristics, management and survival
Source: PLoS One. 2019 Sep 6;14(9):e0220246. doi: 10.1371/journal.pone.0220246 (PMC6730913; doi:10.1371/journal.pone.0220246)

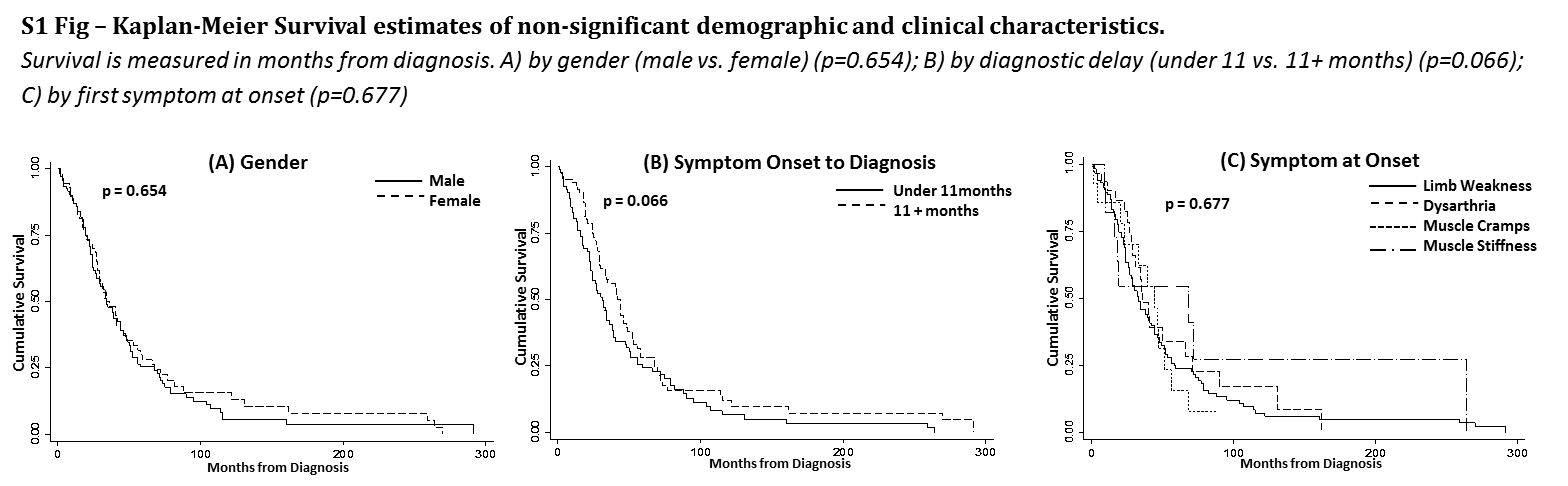

Supplement: S1 Fig — Survival is measured in months from diagnosis. A) by gender (male vs. female) (p = 0.654); B) by diagnostic delay (under 11 vs. 11+ months) (p = 0.066); C) by first symptom at onset (p = 0.677). (DOCX) [file pone.0220246.s003.docx]
